# Supplementary material for: Pyk2 modulates hippocampal excitatory synapses and contributes to cognitive deficits in a Huntington's disease model
Source: Nat Commun. 2017 May 30;8:15592. doi: 10.1038/ncomms15592 (PMC5459995; doi:10.1038/ncomms15592)
Supplement: Supplementary Information — Supplementary Figures and Supplementary Table [file ncomms15592-s1.pdf]

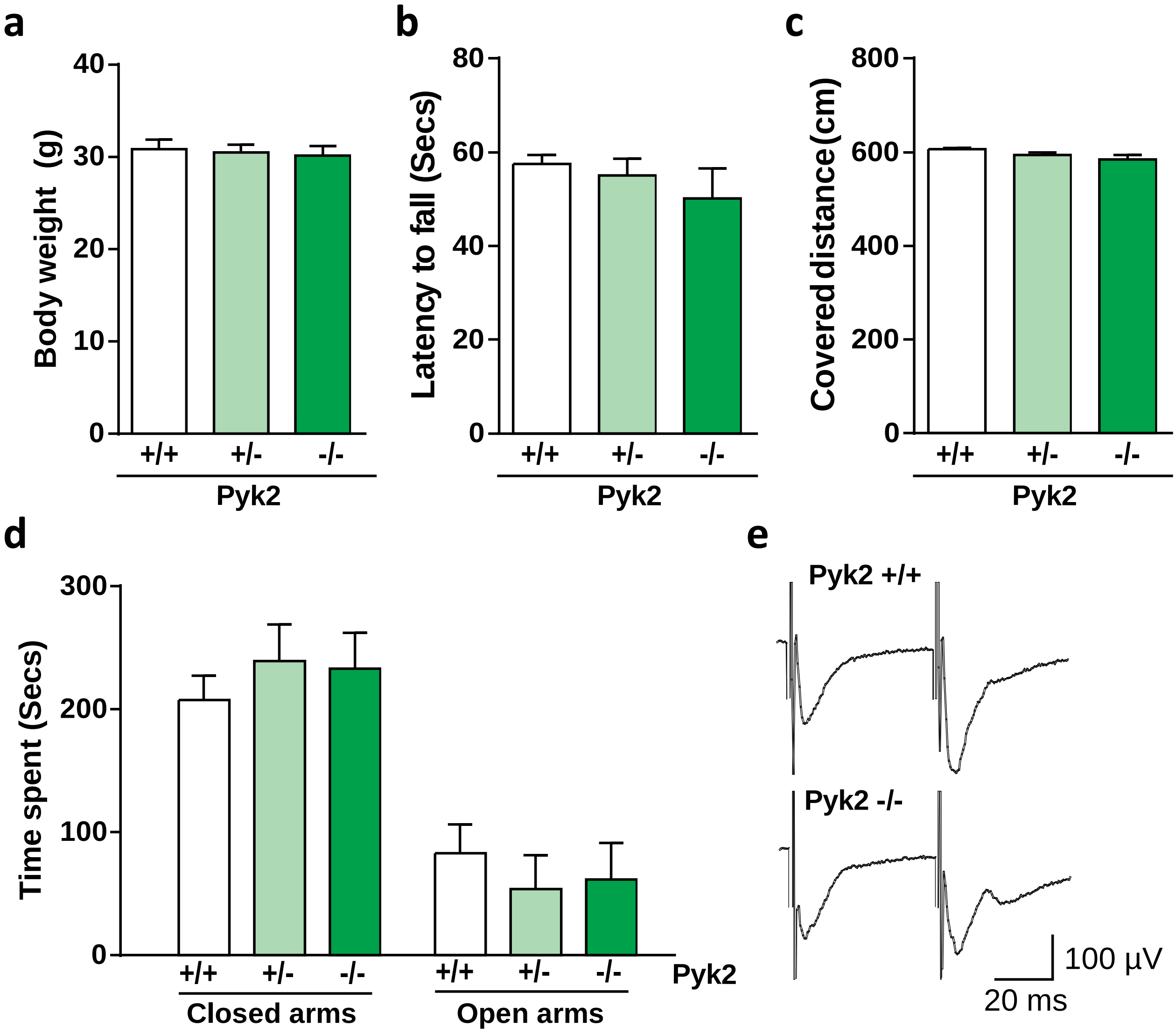

Supplementary Figure 1. General behavioral characterization of Pyk2 deficient mice. (**a**) Body weight and (**b**) muscular strength (measured by the wire hanging test) were monitored in Pyk2$^{+/+}$, Pyk2$^{+/-}$ and Pyk2$^{-/-}$ mice. Kruskal-Wallis test **a**, 0.36, $p = 0.834$; **b**, 0.447, $p = 0.80$. (**c**) Locomotor activity was measured in an open field for 15 min. One way ANOVA, $F_{(2, 24)} = 2.8$, $p = 0.08$. (**d**) The elevated plus maze paradigm was used to evaluate anxiety levels in mice of each genotype. Two-way ANOVA, no genotype effect ($F_{(2,48)} = 0.02$, $p = 0.97$. Values are means + SEM, 7-12 mice/genotype. (**e**) Sample traces of post-synaptic responses used to measure paired-pulse ratio in **Fig. 1e**.

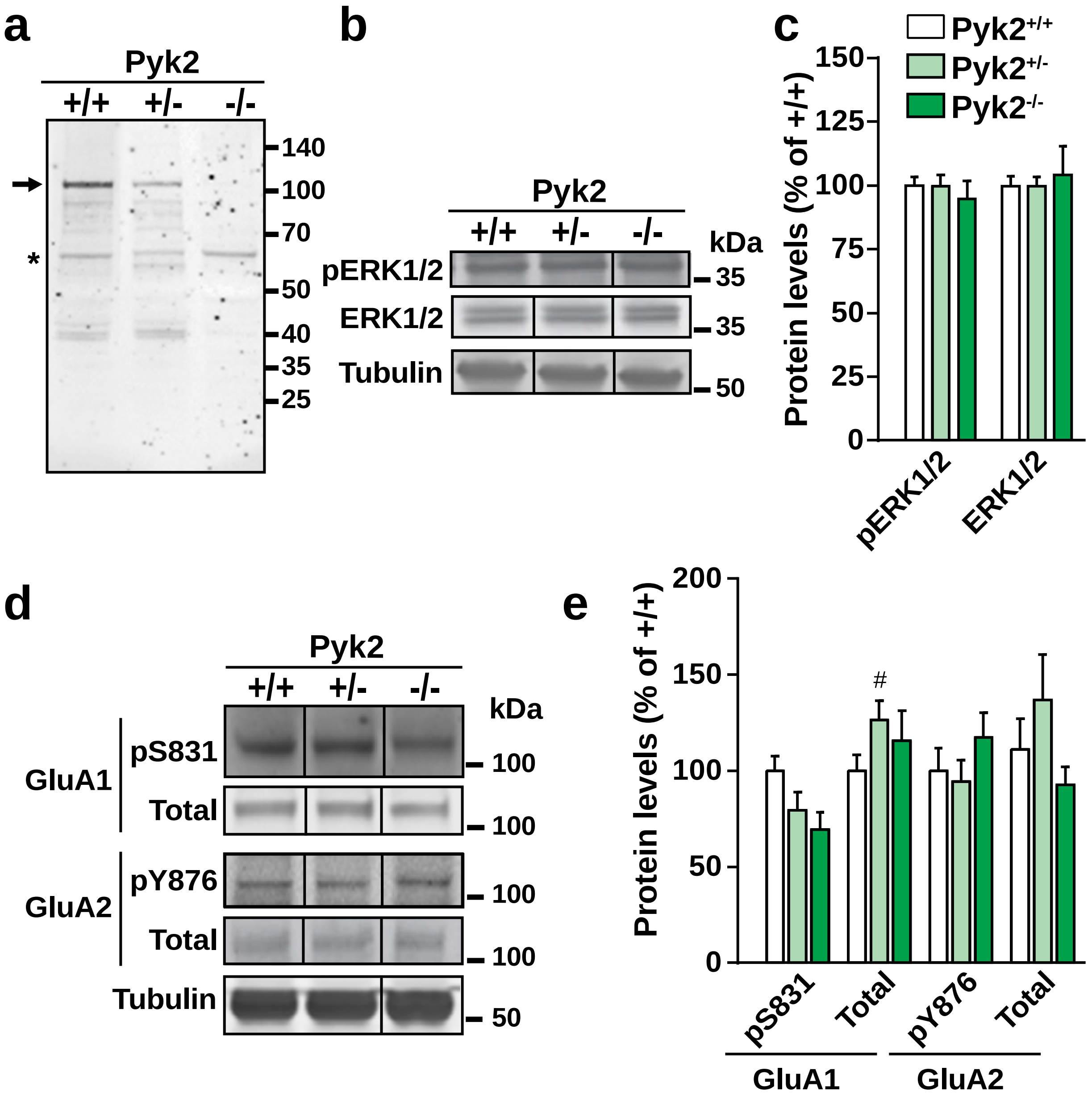

Supplementary Figure 2. Synaptic protein levels in the hippocampus of Pyk2 deficient mice. (**a**) Immunoblotting for Pyk2 with an antibody reacting with the N-terminal region. Pyk2 position is indicated by an arrow and a non-specific cross-reacting band by an asterisk. No additional fragment corresponding to a putative N-terminal domain of Pyk2 was detected in the Pyk2 KO sample. (**b**) Immunoblotting for total ERK1/2 and phosphoERK1/2, and α-tubulin as a loading control in the hippocampus of Pyk2$^{+/+}$, Pyk2$^{+/-}$, and Pyk2$^{-/-}$ mice. (**c**) Quantification of results as in **b**. (**d**) Immunoblotting for phosphorylated forms and total GluA1 and GluA2 as indicated in Pyk2$^{+/+}$, Pyk2$^{+/-}$, and Pyk2$^{-/-}$ mice. (**e**) Quantification of results as in **d**. Values are means + SEM (n = 7-12/genotype). See **Supplementary Table 1** for statistical analysis. # p<0.05 as compared to wt. Mice were 3-month-old. Uncropped blots for **b** and **d** are shown in **Supplementary Figures 12** and **13**, respectively.

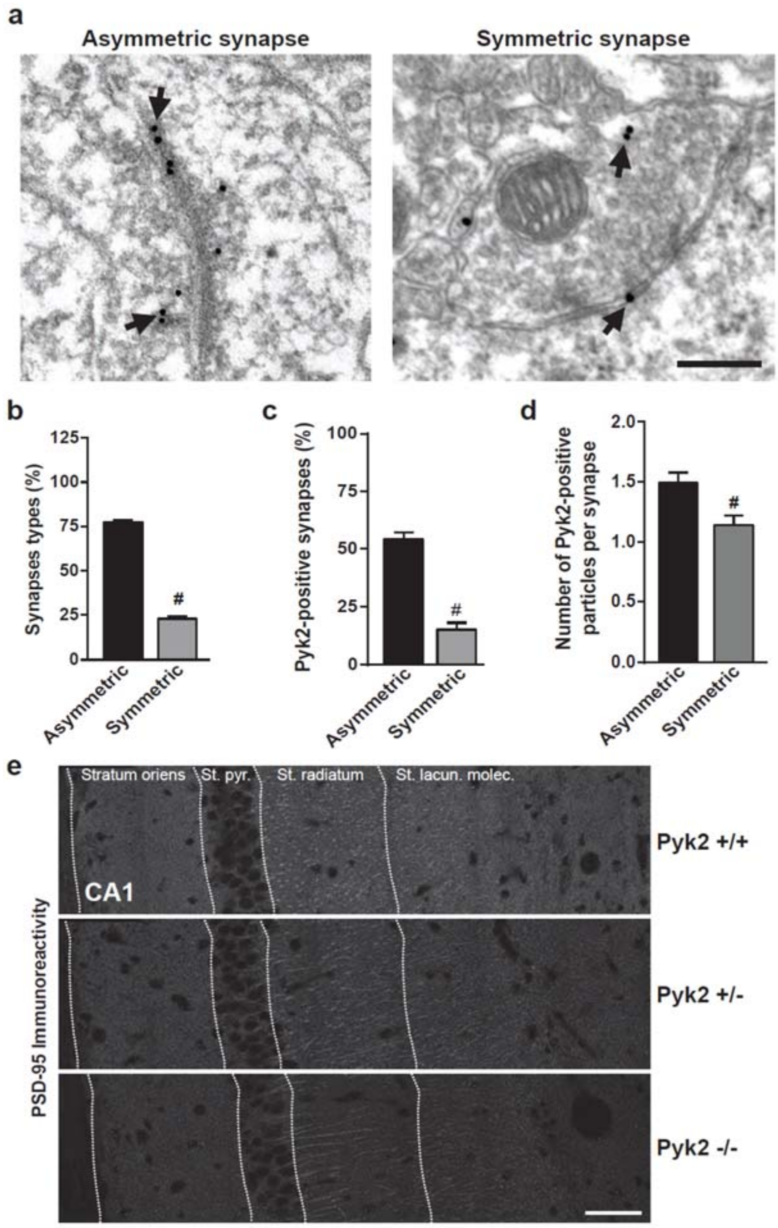

Supplementary Figure 3. Ultrastructural localization of Pyk2 at synapses and distribution of PSD-95 in CA1. (**a**) Electron microscopy images at 24 500 x in CA1 *stratum radiatum* of wild type mice were used to localize Pyk2-like immunoreactivity in asymmetric and symmetric synapses. Representative images of asymmetric synapse (left) and symmetric (right) synapse with Pyk2 immunoreactivity revealed by secondary immunogold particles. (**b**) Quantification of asymmetric and symmetric synapses in the *stratum radiatum* expressed as a percent of total number of synapses in the fields examined. Two-tailed Mann and Whitney's test, $p = 0.03$. (**c**) Percentage of asymmetric and symmetric synapses containing Pyk2-positive particles, 2-tailed Mann and Whitney's test, $p = 0.03$. (**d**) Number of Pyk2 immunogold particles in post-synaptic compartments of asymmetric and symmetric Pyk2-positive synapses (total of 136 synapses counted). Two-tailed Mann and Whitney's test, $p = 0.02$. **b-d**, Values are means + SEM (20-30 images from 4 Pyk2$^{+/+}$ mice). # $p < 0.05$ as compared to asymmetric synapses. (**e**) Mosaic pictures of PSD-95 immunoreactivity (as in **Fig. 3d**) along the entire depth of CA1 from wild type (+/+) heterozygous (+/-) and homozygous (-/-) Pyk2 knockout mice. The limits of layers is indicated (dotted line), St. pyr., *stratum pyramidale*, St. lacun. molec., *stratum lacunosum moleculare*. Scale bar in **a**, 0.2 µm, in **e**, 50 µm.

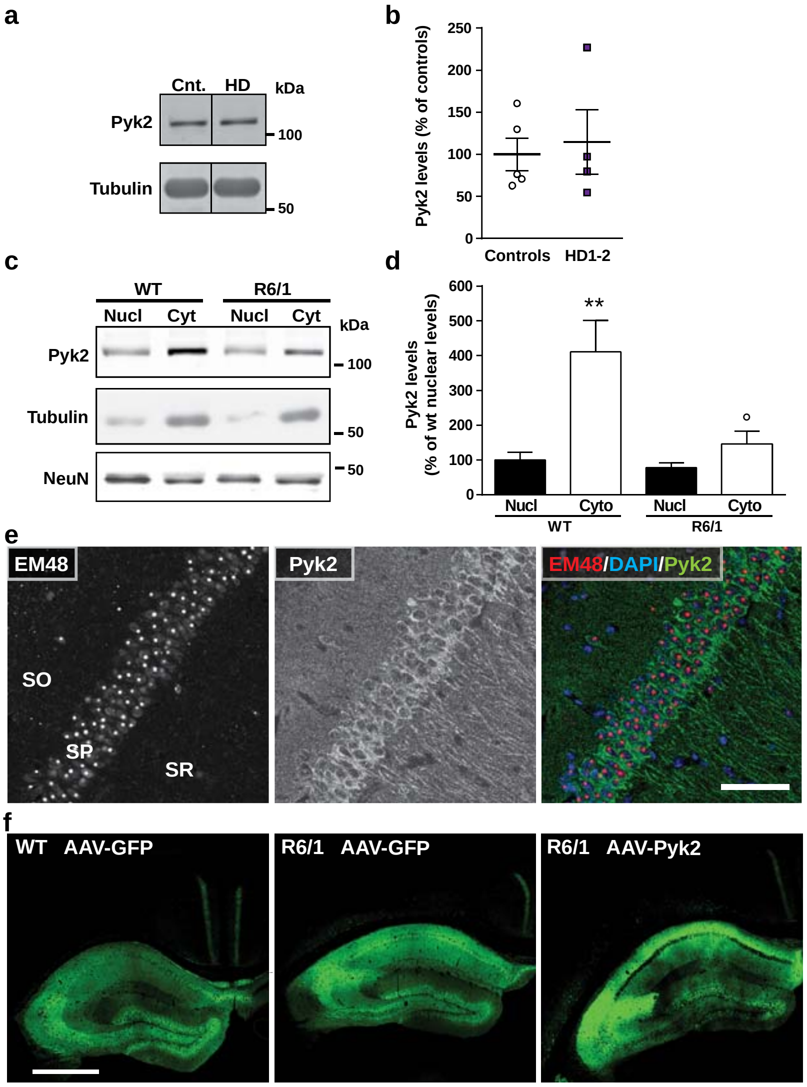

Supplementary Figure 4. Pyk2 in low grade HD patients and in R6/1 mice. (**a**) Hippocampal post-mortem samples from human patients grade 1-2 (HD1-2) and controls (Cnt.) were analyzed by immunoblotting for Pyk2 and α-tubulin as a loading control. Molecular weight marker positions are indicated in kDa. (**b**) Densitometric quantification of results as in **a** expressed as a percentage of the mean in controls (controls, n = 5, HD1-2, n = 4). Student's t test, $t_7 = 0.37$, n.s. (**c**) Pyk2 is not sequestered in intra-nuclear inclusions and is specifically reduced in the cytosol in R6/1mice. Immunoblotting for total Pyk2, α-tubulin, and NeuN as controls in nuclear (Nucl) and cytoplasmic (Cyto) fractions of the hippocampus from wild type (WT) and R6/1 mice. (**d**) Quantification of results as in **c** for Pyk2. Values are means + SEM. Two-way ANOVA: localization effect, $F_{(1,16)}=9.93$, p=0.006, genotype effect, $F_{(1,16)}=5.66$, p=0.03, interaction $F_{(1,16)}=4.04$, p=0.06. Holm-Sidak's test, cytoplasm vs nucleus, ** p<0.01, R6/1 vs WT, ° p<0.05. (**e**) Confocal microscopy images of double immunofluorescence for EM48 (red) and Pyk2 (green) in CA1 of R6/1 mice. SO, *stratum oriens*, SP, *stratum pyramidale*, SR, *stratum radiatum*. Scale bar, 25 µm. (**f**) GFP fluorescence distribution in the hippocampus and adjacent cortex of WT and R6/1 mice which received intra-hippocampal injections of AAV expressing GFP (AAV-GFP) or Pyk2 and GFP (AAV-Pyk2). Stitched confocal images. Scale bar 200 µm. Uncropped blots for **a** and **c** are shown in **Supplementary Figures 14** and **15**, respectively.

Supplementary Figure 5. Full-size immunoblots related to Fig. 2a. Red boxes indicate regions shown in the corresponding Figure. Molecular weight markers positions (kDa).

.

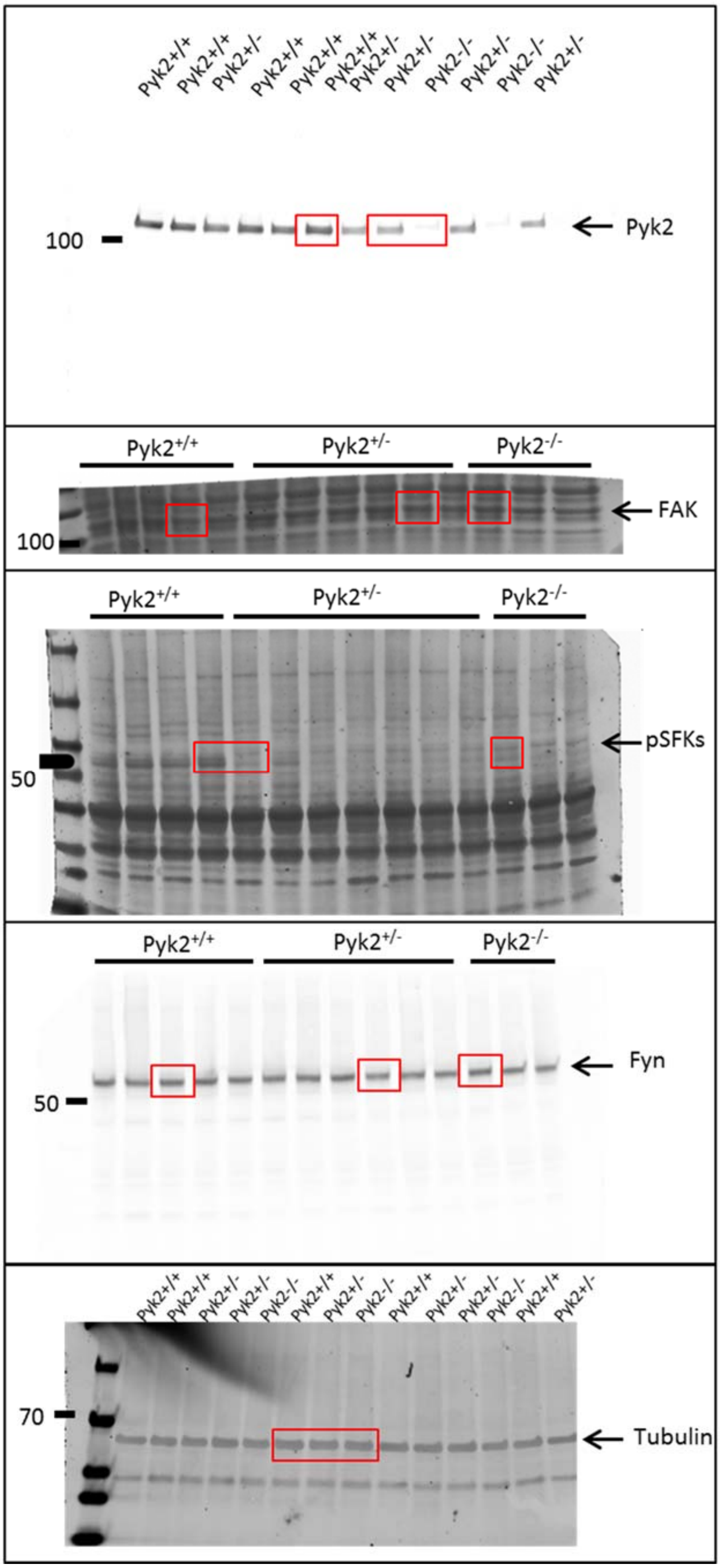

Supplementary Figure 6. Full-size immunoblots related to Fig. 2c. Red boxes indicate regions shown in the corresponding Figure. Molecular weight markers positions (kDa).

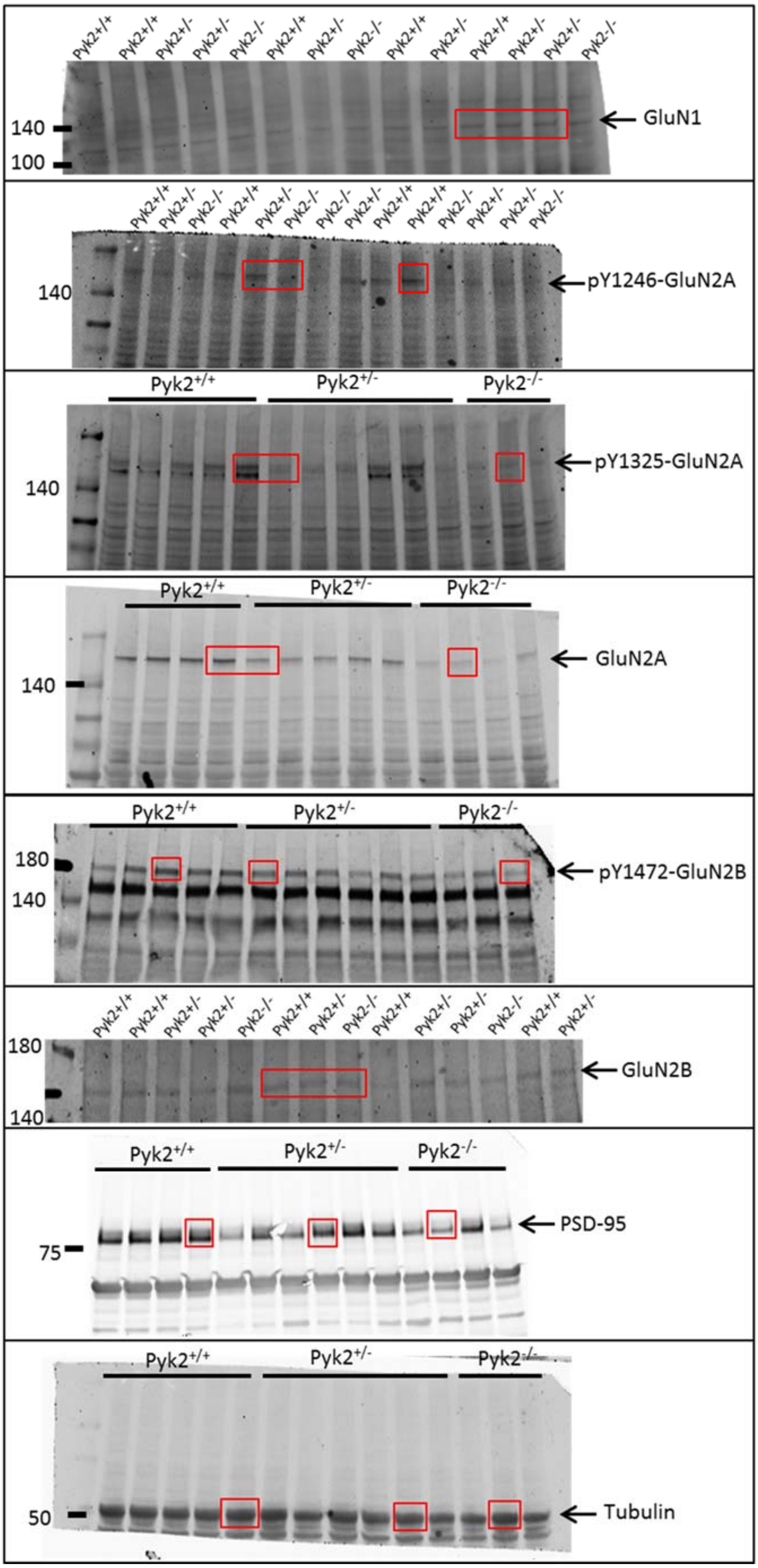

Supplementary Figure 7. Full-size immunoblots related to Fig. 2e. Red boxes indicate regions shown in the corresponding Figure. Molecular weight markers positions (kDa).

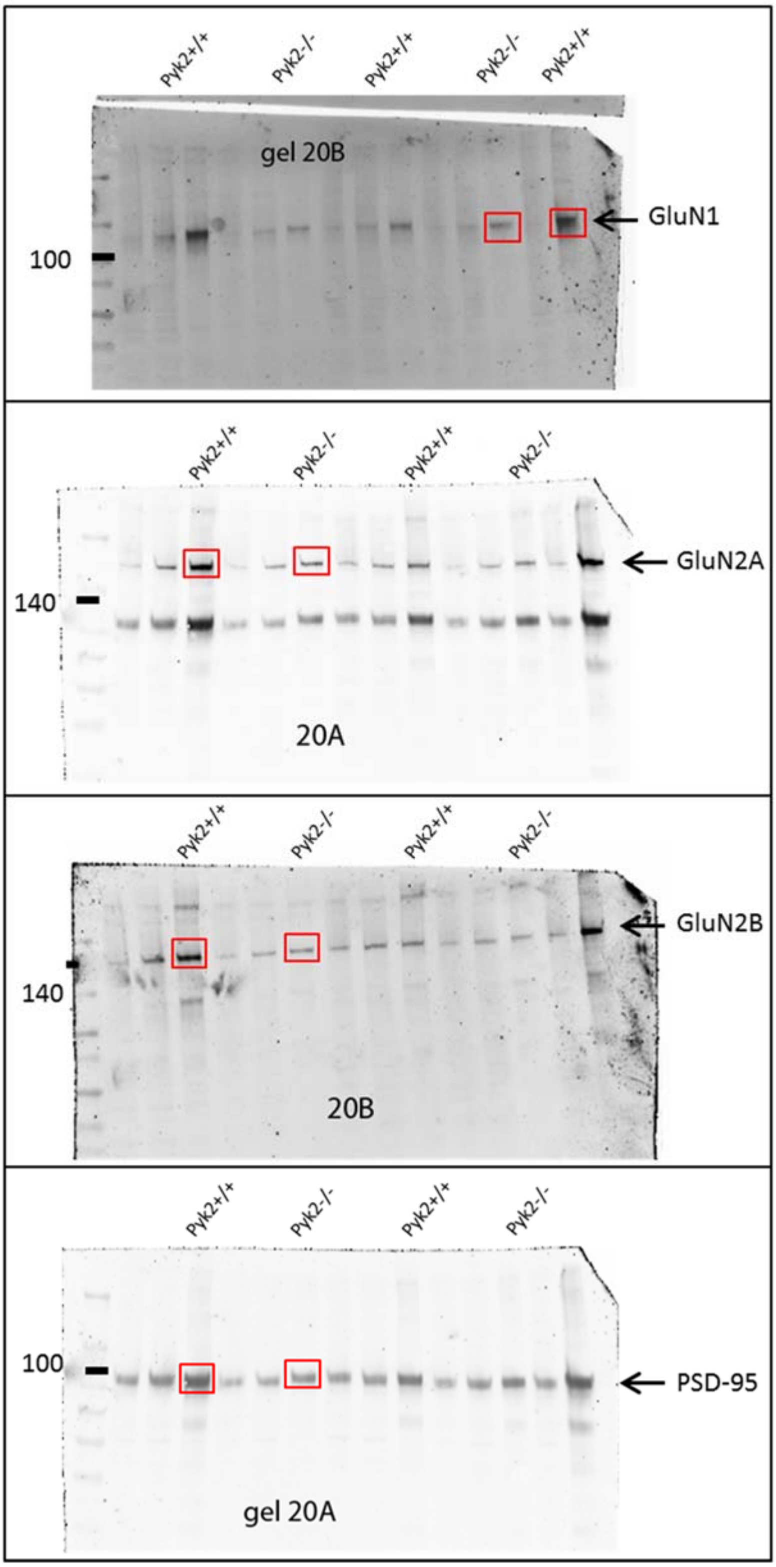

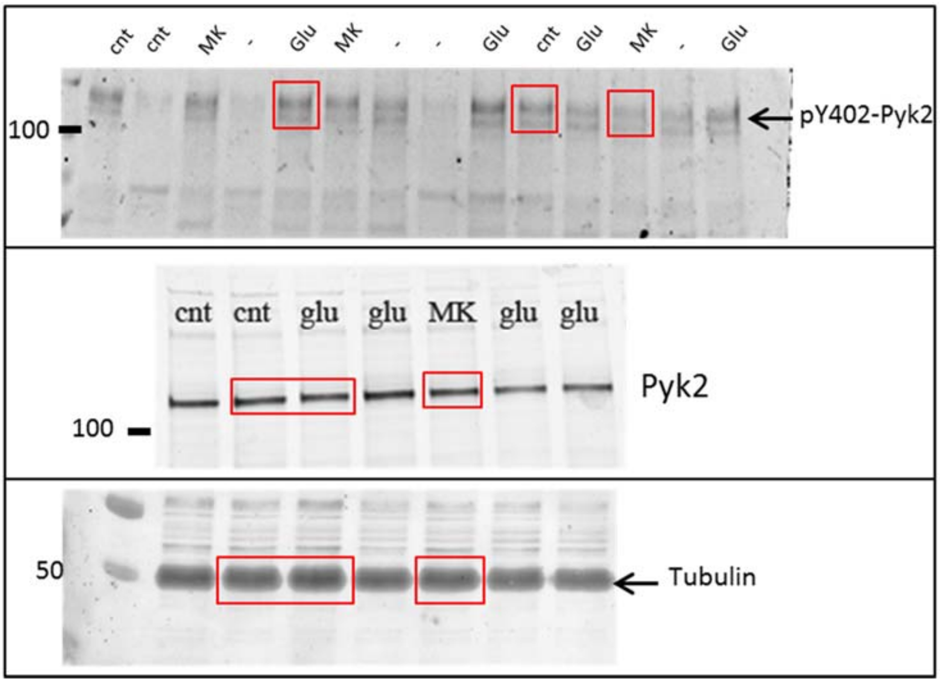

Supplementary Figure 8. Full-size immunoblots related to Fig. 5a. Red boxes indicate regions shown in the corresponding Figure. Molecular weight markers positions (kDa).

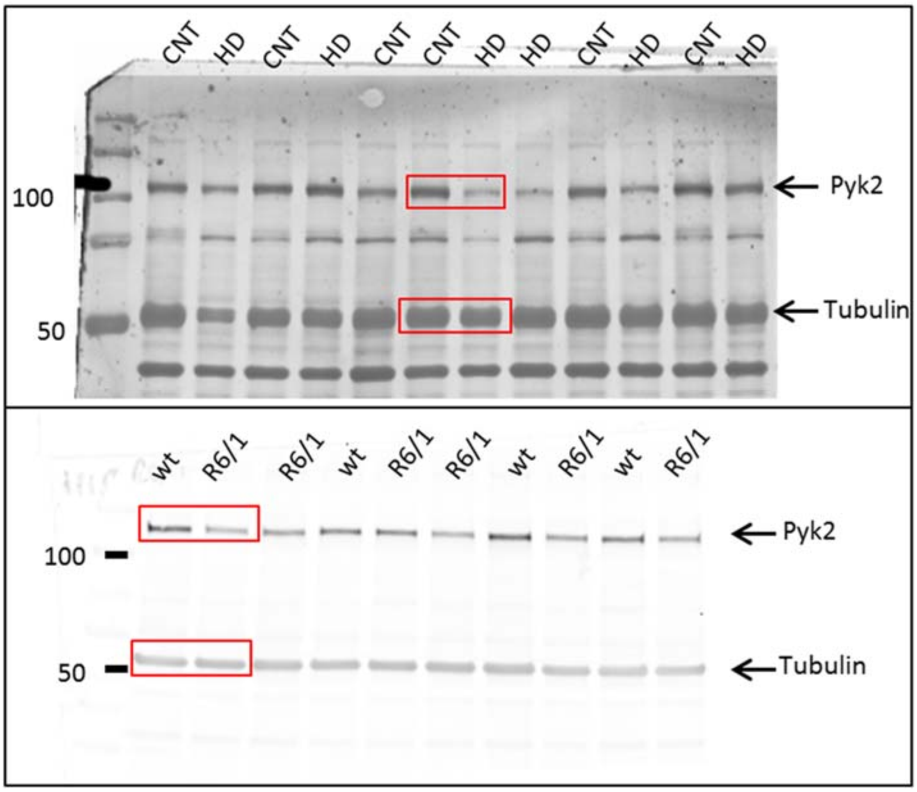

Supplementary Figure 9. Full-size immunoblots related to Fig. 7a. Red boxes indicate regions shown in the corresponding Figure. Molecular weight markers positions (kDa).

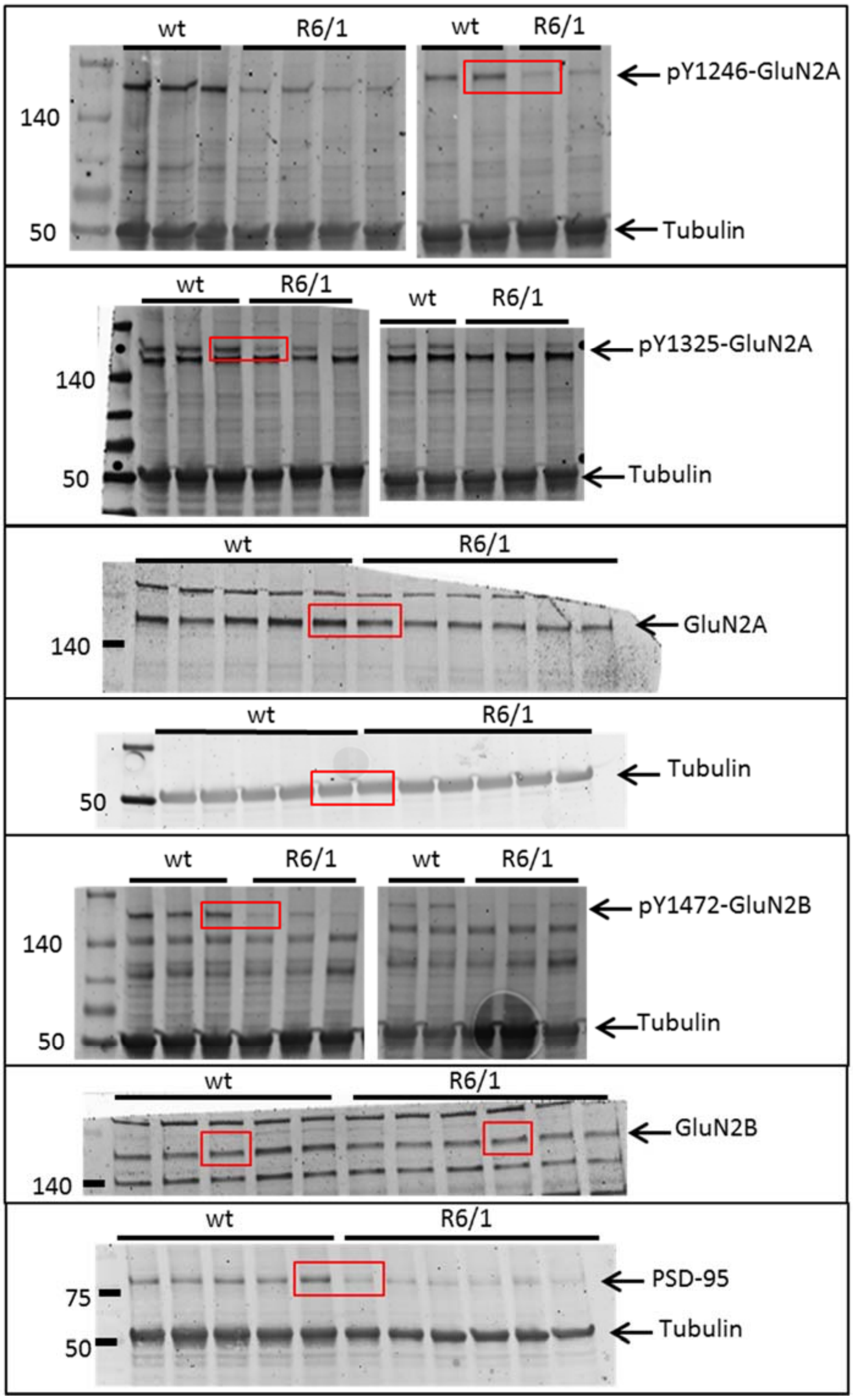

Supplementary Figure 10. Full-size immunoblots related to Fig. 7d. Red boxes indicate regions shown in the corresponding Figure. Molecular weight markers positions (kDa).

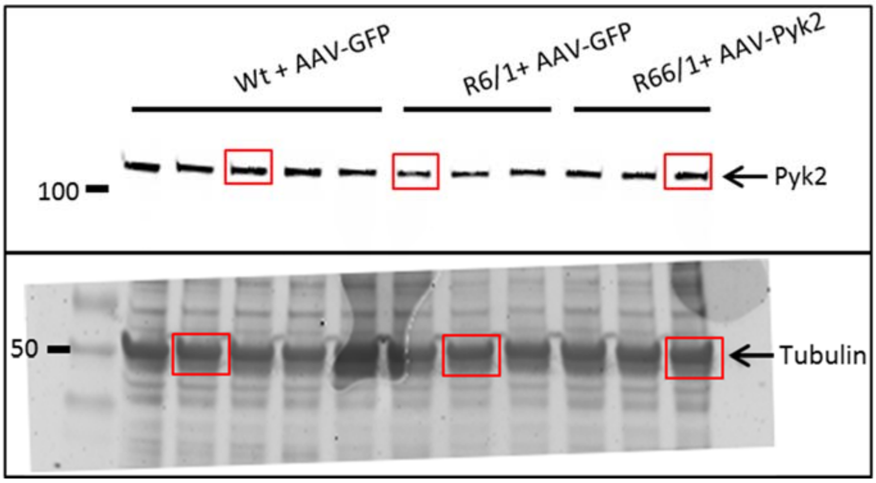

Supplementary Figure 11. Full-size immunoblots related to Fig. 8a. Red boxes indicate regions shown in the corresponding Figure. Molecular weight markers positions (kDa).

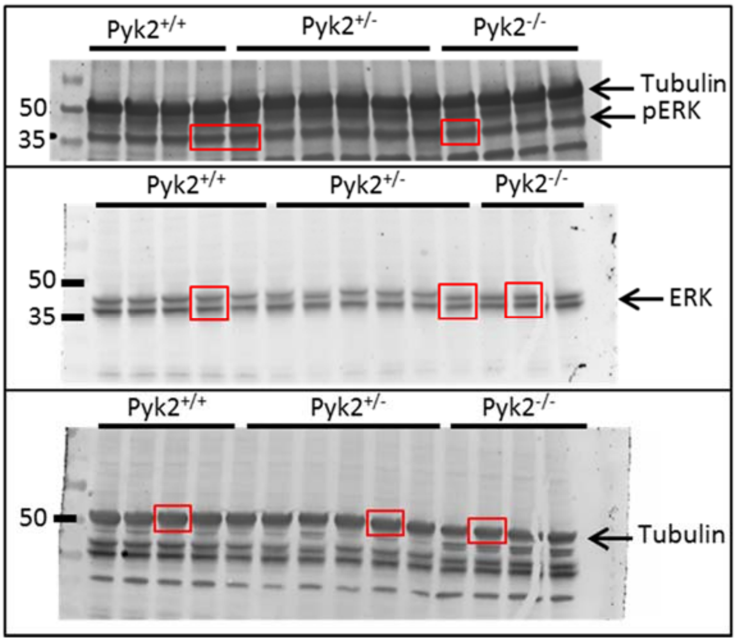

Supplementary Figure 12. Full-size immunoblots related to Supplementary Figure 2b. Red boxes indicate regions shown in the corresponding Figure. Molecular weight markers positions (kDa).

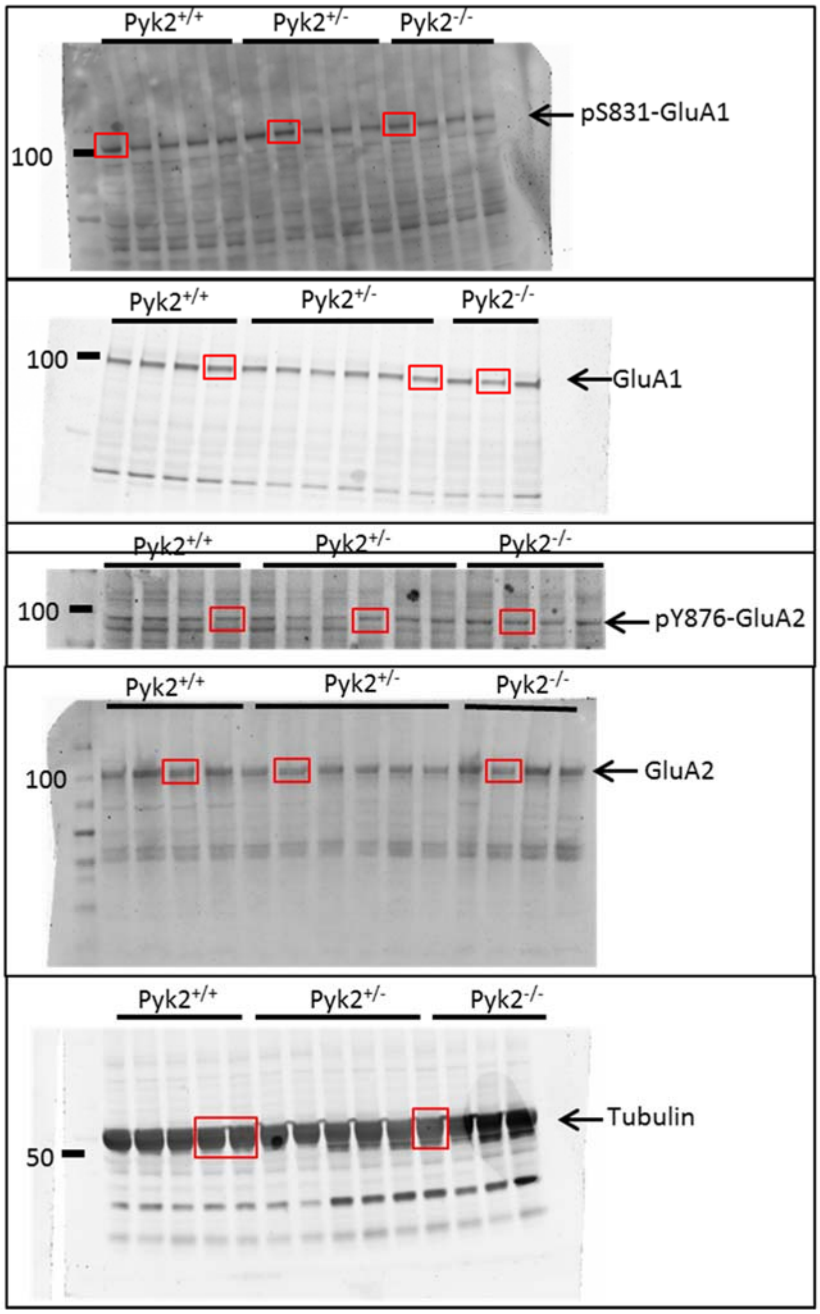

Supplementary Figure 13. Full-size immunoblots related to Supplementary Figure 2d. Red boxes indicate regions shown in the corresponding Figure. Molecular weight markers positions (kDa).

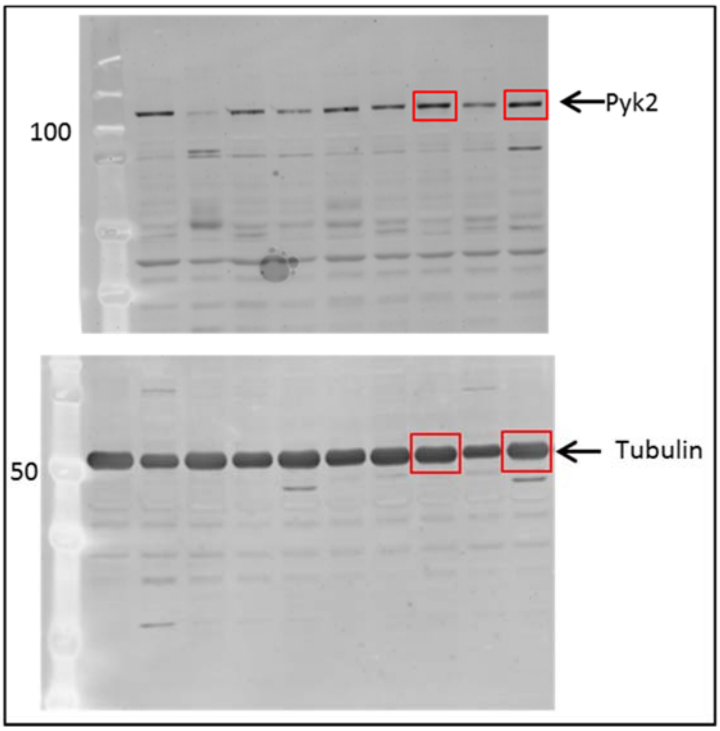

Supplementary Figure 14. Full-size immunoblots related to Supplementary Figure 4a. Red boxes indicate regions shown in the corresponding Figure. Molecular weight markers positions (kDa).

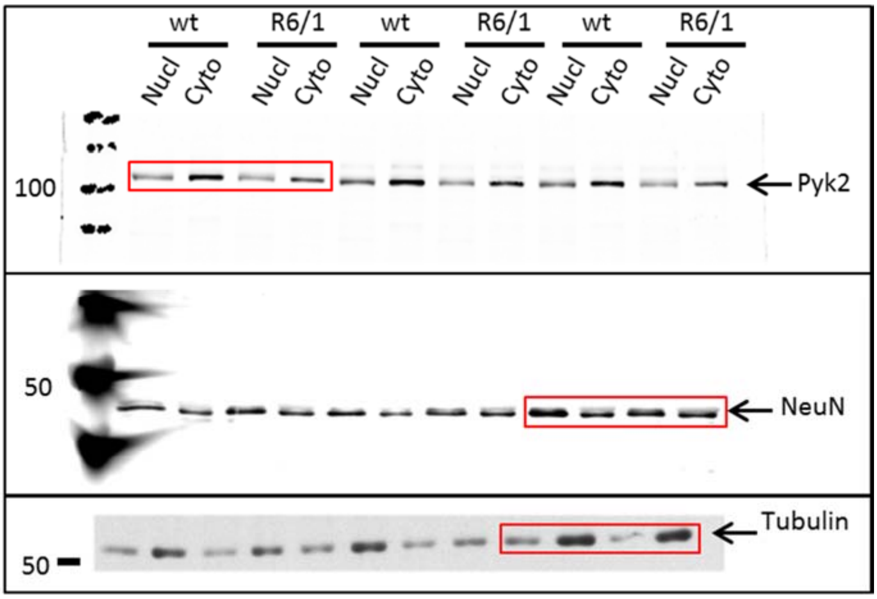

Supplementary Figure 15. Full-size immunoblots related to Supplementary Figure 4c. Red boxes indicate regions shown in the corresponding Figure. Molecular weight markers positions (kDa).

Supplementary Table 1: Statistical analysis values for Fig. 2 and Suppl. Fig. 2

				Normality test								Dunn's test						Holm Sidak's test					
	n			d'Agostino Pearson			Shapiro Wilk			Kruskal Wallis		+/- vs +/+	-/- vs +/+	-/- vs +/-	One way ANOVA			+/- vs +/+		-/- vs +/+		-/- vs +/-	
	+/+	+/-	-/-	+/+	+/-	-/-	+/+	+/-	-/-	KW	p	p	p	p	DF1, DF2	F	p	t	p	t	p	t	p
ERK	9	12	7	Yes	Yes	nts	Yes	Yes	Yes						2, 25	0.15	ns						
pERK	9	12	7	Yes	Yes	nts	Yes	Yes	Yes						2, 25	0.31	ns						
FAK	9	10	4	Yes	Yes	nts	Yes	Yes	nts						2, 20	0.31	ns						
Fyn	9	99	8	Yes	Yes	Yes	Yes	Yes	Yes						2, 23	1.13	ns						
pY418Fyn/SFK	9	11	8	Yes	Yes	Yes	Yes	Yes	Yes						2, 25	15.84	$<10^{-4}$	4.07	$<10^{-3}$	5.42	$<10^{-4}$	1.74	ns
GluA1	9	9	8	Yes	Yes	No	Yes	Yes	No	6.13	0.047	ns	*	ns									
GluA1 pS831	9	12	7	Yes	Yes	nts	Yes	Yes	Yes						2, 25	2.60	ns						
GluA2	9	10	8	Yes	Yes	Yes	Yes	Yes	Yes						2, 24	1.43	ns						
GluA2 pY876	9	11	7	No	No	Yes	No	No	nts	1.93	0.38												
GluN1	9	11	7	Yes	Yes	nts	Yes	Yes	Yes						2, 24	0.40	ns						
GluN2A	9	11	7	Yes	Yes	nts	Yes	No	Yes	17.5	0.0002	ns	****	ns									
pY1246-GluN2A	9	11	7	Yes	Yes	nts	No	Yes	Yes	6.64	0.036	ns	*	ns									
pY1325-GluN2A	9	11	7	Yes	No	nts	Yes	No	Yes	15.40	0.0005	ns	***	ns									
GluN2B	8	11	7	Yes	No	nts	Yes	No	Yes	3.16	0.21												
GluN2B pY1472	8	11	7	Yes	Yes	nts	Yes	Yes	Yes						2, 23	13.93	$<10^{-3}$	3.00	<0.05	5.27	$<10^{-4}$	2.75	<0.05
PSD-95	9	12	7	Yes	Yes	nts	Yes	Yes	Yes						2, 25	9.51	$<10^{-3}$	1.92	ns	4.35	$<10^{-3}$	2.83	<0.05
Pyk2	6	4	4	nts	nts	nts	nts	nts	nts						2, 11	156.9	$<10^{-4}$	8.23	$<10^{-4}$	17.67	$<10^{-4}$	8.61	$<10^{-4}$

For each protein or phosphorylated form the number of mice (n) is indicated. Normality was tested with d'Agostino-Pearson and Shapiro-Wilk tests. If the distribution was not normal ($p<0.05$, No), further analysis was done with Kruskal-Wallis (value, KW), and Dunn's test for multiple comparisons. When the distribution was not different from normal (Yes), one-way ANOVA and multiple comparisons with Holm Sidak's test were used. The degrees of freedom (DF1, DF2), the F value, and the corresponding p value are indicated for each ANOVA. ns, not significant, nts, number too small for the test.
